# *PROL1* is essential for xenograft tumor development in mice injected with the human prostate cancer cell-line, LNCaP, and modulates cell migration and invasion

**DOI:** 10.31083/jomh.2021.131

**Published:** 2021-10-14

**Authors:** Amarnath Mukherjee, Augene Park, Kelvin Paul Davies

**Affiliations:** 1 Department of Urology, Albert Einstein College of Medicine, Bronx, NY 10461, USA; 2 Department of Physiology and Biophysics, Albert Einstein College of Medicine, Bronx, NY 10461, USA

**Keywords:** Cell motility, Cell invasiveness, Opiorphin, *PROL1*, Prostate cancer

## Abstract

**Background and objective:**

A growing body of literature suggests modulated expression of members of the opiorphin family of genes (*PROL1*, *SMR3A* and *SMR3B*) is associated with cancer. Recently, overexpression of *PROL1* was shown to be associated with prostate cancer, with evidence of a role in overcoming the hypoxic barrier that develops as tumors grow. The primary goal of the present studies was to support and expand evidence for a role of *PROL1* in the development and progression of prostate cancer.

**Material and methods:**

We engineered knock-out of the opiorphin gene, *PROL1,* in LNCaP, an androgen-sensitive, human prostate cancer derived, cell-line. Using xenograft assays, we compared the ability of injected LNCaP *PROL1* knock-out cell-lines to develop tumors in both castrated and intact male mice with the parental LNCaP and LNCaP *PROL1* overexpressing cell-lines. We used RNAseq to compare global gene expression between the parental and LNCaP *PROL1* knock-out cell-lines. Wound closure and 3D spheroid invasion assays were used to compare cell motility and migration between parental LNCaP cells and LNCaP cells overexpressing of *PROL1*.

**Results:**

The present studies demonstrate that LNCaP cell-lines with consisitutive knock-out of *PROL1* fail to develop tumors when injected into both castrated and intact male mice. Using RNAseq to compare global gene expression between the parental and LNCaP *PROL1* knock-out cell-lines, we confirmed a role for *PROL1* in regulating molecular pathways associated with angiogenesis and tumor blood supply, and also identified a potential role in pathways related to cell motility and migration. Through the use of wound closure and 3D spheroid invasion assays, we confirmed that overexpression of *PROL1* in LNCaP cells leads to greater cell motility and migration compared to parental cells, suggesting that *PROL1* overexpression results in a more invasive phenotype.

**Conclusion:**

Overall, our studies add to the growing body of evidence that opiorphin-encoding genes play a role in cancer development and progression. *PROL1* is essential for establishment and growth of tumors in mice injected with LNCaP cells, and we provide evidence that *PROL1* has a possible role in progression towards a more invasive, metastatic and castration resistant prostate cancer (PrCa).

## 1. Introduction

In the majority of countries throughout the world, prostate cancer (PrCa) is the most common cancer affecting men; in 2018 there were approximately 1.3 million PrCa patients and 400,000 associated deaths [1]. Although the prognosis for patients with localized PrCa is good, metastatic, castration-resistant PrCa is invariably lethal [2]. Research aimed at increasing our understanding of the mechanisms and factors involved in the development of metastatic castration-resistant tumors has the potential to identify novel therapeutic strategies for PrCa.

In this regard, a growing body of evidence associates modulated expression of members of the opiorphin gene family (represented by *PROL1, SMR3A and SMR3B*) with several cancers [3–10], including a recently identified association between upregulated expression of *PROL1* and PrCa [11]. The opiorphin genes encode peptides which act as potent endogenous neutral endopeptidase (NEP) inhibitors [12]. Opiorphin genes have previously been reported to act as master regulators of the hypoxic response in smooth muscle cells [13, 14], and *PROL1* has recently been shown to regulate the expression of genes involved in the hypoxic response in PrCa cell-lines. Therefore, it has been suggested that *PROL1* may play a role in overcoming the “hypoxic barrier”, which results develops in the initial phase of tumor growth when uncontrolled cell proliferation often exceeds the ability to satisfy the oxygen demand from the preexisting blood vessel [15, 16]. This usually occurs when the tumor exceeds a diameter of approximately 1 mm [17–19]. Overcoming the hypoxic barrier allows the tumor to develop, with increased invasion of local tissue and potential for metastasis to other parts of the body [20, 21]. In addition, activation of the hypoxic response pathways has been associated with malignant progression towards castration resistant PrCa [22–24].

The primary goal of the present studies was to support and expand evidence for a role of *PROL1* in the development and progression of PrCa. We generated a *PROL1* knock-out LNCaP cell-line (LNCaP-ProL1–) and compared its ability to generate tumors when injected into mice with the parent (LNCaP) and *PROL1* overexpressing (LNCaP-ProL1+) cell-lines. Global gene expression analysis confirmed a role for *PROL1* in regulating molecular pathways associated with angiogenesis and tumor blood supply, but also identified a potential role in pathways related to cell motility and migration. We confirmed that overexpression of *PROL1* in LNCaP-ProL1+ results in increased cell motility and migration, suggesting a role of *PROL1* in both growth and progression of PrCa.

## 2. Materials and methods

### 2.1 Generation and maintenance of cell-lines (LNCaP, LNCaP-ProL1+, LNCaP-ProL1–)

LNCaP clone FGC (NCI-PBCF-CRL1740 (LNCaP Clone FGC)/ATCC® CRL-1740*^™^*; hereafter termed LNCaP) was obtained from the American Type Culture Collection (ATCC, Rockville, MD, USA). The generation and characterization of LNCaP cells overexpressing *PROL1* (LNCaP-ProL1+) has been previously described [11]. *PROL1* knockout cell-lines (LNCaP-ProL1–) were generated using a commercially available *PROL1* CRISPR knockout kit (Origene Technologies, Rockville, MD, USA); gRNA sequence GGACTTGGTGGAACCCATCT), according to manufacturers’ protocol. Following antibiotic selection, it was confirmed that each colony was expressing GFP before pooling colonies and confirming knock-out of *PROL1* across the non-clonal population. Control cell-lines for both LNCaP-ProL1+ and LNCaP-ProL1– were generated using a lentiviral control vector (Catalog #: PS100093V, Origene Technologies) and a scramble CRISPR control vector (Catalog #: GE100003, Origene Technologies), respectively. Cell-lines were maintained in Roswell Park Memorial Institute (RPMI) 1640 medium (Invitrogen, Carlsbad, CA, USA) with 10% Fetal Bovine Serum (FBS, Thermo Fisher Scientific, Waltham, MA, USA), supplemented with 100 U/mL penicillin G and 100 ng/mL streptomycin (Invitrogen). In studies investigating the growth of cells in androgen depleted media the FBS was replaced with charcoal-stripped FBS (Sigma Aldrich, Burlington MA, USA).

All cell-lines were passaged on reaching 70% confluency (approximately 2 to 3-day intervals) using a 0.25% Trypsin-EDTA solution (Thermo Fisher Scientific). Cell morphology and viability were monitored by microscopic observation using the CellTiter 96® AQueous One Solution Cell Proliferation Assay (MTS) from Promega (Madison, WI, USA) and regular Mycoplasma testing was performed (Universal Mycoplasma Detection Kit; ATCC). Growth rate was calculated as the average doubling time in cell number over a 24-hour period when cells were in the logarithmic growth phase (performed in triplicate).

### 2.2 Mouse xenograft studies

Note: All animal studies were conducted in accordance with the Animal (Scientific Procedures) Act 1986 and approved by the Institutional Animal Care and Use Committee (IACUC) of Albert Einstein College of Medicine (protocol 20170801).

The mouse xenograft studies used the same methods and procedures as previously described [11]. Castrated and intact male nude mice were injected with 2.5 × 10^6^ LNCaP-ProL1+, LNCaP-ProL1– and LNCaP cells and tumor size determined twice a week. The number of animals in each group are described in the Figure legends. Tumors were measured twice a week. Animals were euthanized 13-weeks post-innoculation, or if they developed any tumors >1000 mm^3^, through inhalation of CO_2_ to effect.

### 2.3 Wound healing assay

Wound healing assays were performed as previously described [25]. Briefly, cells were cultured in a 60 mm culture dish until reaching 90%–100% confluency at which point a scratch was created using a p200 pipette tip. Healing was monitored under an Olympus IX71 microscope equipped with Olympus DP72 camera. The distance between cell borders were determined using CellSens Standard imaging software (Olympus Life Science, Waltham, MA, USA).

### 2.4 Spheroid tumor invasion assay

The spheroid tumor invasion assay was adapted from a previously described method [26]. Briefly, 500–1000 cells were taken from culture and suspended in 20 *μ*L media, which was then pipetted onto the inner surface of 10 cm plate lid (40 drops in total). The lid was placed on a cell culture dish containing PBS and placed in an incubator. Spheroids were generated after approximately 3 days of culture and transferred into a microcentrifuge tube containing a mix of 100 *μ*L Matrigel (Corning Life Sciences, Teterboro, NJ, USA) and 100 *μ*L 3 mg/mL rat tail collagen I (Thermo Fisher Scientific). This mixture was embedded to the wells of a 24 well-plate (pre-treated with Matrigel) along with 1 mL cell culture media. After two weeks, invasion of the spheroids was determined through optical microscopy. Spheroids were determined to be invasive if a 2D layer of cells was observed growing around the spheroid.

### 2.5 RNA Isolation, quantitative RT-PCR and RNAseq

RNA was isolated from cell-lines using the RNeasy Plus Mini Kit (Qiagen, Hilden, Germany) and used to determine expression levels of *PROL1* by quantitative-RT-PCR as previously described [11]. RNA was also sent to a commercial vendor (Novogene Corp., Sacramento, CA, USA) for performance of RNA-seq analysis of global gene expression as previously described. Similar criteria were used to identify differentially expressed genes (DEG) between LNCaP and LNCaP-ProL1– cell-lines as described in the analysis of DEG in LNCaP compared to LNCaP-ProL1+ cell-lines as previously described (i.e., cut-off criteria for differentially expressed genes was >1 Log2FoldChange or <–1 Log2FoldChange in gene expression with a *p*-value < 0.01 [11]). Gene ontology (GO) annotation analysis of DEG was performed using online analysis tools available from the database for annotation, visualization and integrated discovery (DAVID, vers 6.8, *Homo sapien* GOTERM_GO_Direct database [27, 28]).

### 2.6 Statistical analyses

Statistical analyses were performed using either Microsoft Excel (Microsoft, Seattle, WA, USA) or Prism 8.2. (Graph-Pad Software, Inc., La Jolla, CA, USA). To determine statistical significance of two group comparisons, unpaired, two-tailed *t*-tests were performed, and results reported in Tables and Figures. Error bars represent standard deviation of mean (as described in figure legends).

## 3. Results

### 3.1 Characterization of cell-lines

Using qt-RT-PCR we confirmed knockout of *PROL1* in our genetically engineered LNCaP cell-line (LNCaP-ProL1–). *PROL1* was below the limit of detection in this assay. Prior studies have shown that in the LNCaP cell-line engineered to overexpress LNCaP (LNCaP-ProL1+) there is >28,560-fold overexpression of *PROL1* relative to LNCaP [11]. Under normal culture conditions (10% FBS), although relative to LNCaP there was a trend for the doubling time to increase when *PROL1* was overexpressed, and a trend for doubling time to increase with *PROL1* knockout, this did not reach a significance of *p* < 0.005 (Table 1, Supplementary Fig. 1). However, in media in which FBS was replaced by charcoal-stripped FBS, there was a significant decrease in the growth rate of all cell-lines (as has been previously observed for growth of androgen sensitive cell-lines [29]). As shown in Table 1, compared to LNCaP (where there was a 3.7-fold decrease in growth rate), the overexpression of *PROL1* in LNCaP-ProL1+ partly mitigated this effect (a decrease in growth rate of 2.3-fold), whereas the *PROL1* knockout in LNCaP-ProL1– exacerbated the effect (approximately a 9.8-fold decrease in growth rate, respectively).

### 3.2 PROL1 expression is essential for tumor development and growth in xenografted LNCaP cell-lines

Prior studies have shown that intact male mice injected with either LNCaP or LNCaP-ProL1+ cell-lines develop tumors [11, 30]. In contrast, the LNCaP cell-line developed for the present studies, where *PROL1* was knocked-out (LNCaP-ProL1–), was unable to form tumors when injected into intact male mice (*N* = 5).

### 3.3 Global gene expression analysis confirms a role for PROL1 in regulating molecular pathways associated with angiogenesis and tumor blood supply, and identifies a potential role in cell migration

In order to identify molecular pathways regulated by *PROL1* that may be involved in PrCa growth and progression, we compared differentially expressed genes (DEG) between the LNCaP-ProL1– and LNCaP cell-lines (as described under Materials and Methods, Section 2.5). We identified 1563 DEG, of which 891 had upregulated, and 672 downregulated expression in LNCaP-ProL1– versus LNCaP (Supplementary Table 1). In a previous study, using an identical method of analysis, 1110 DEG were identified when *PROL1* was overexpressed in LNCaP cells [11]. As shown in Supplementary Table 2, 312 DEG were common between the two analyses (representing 20% of the total DEG identified in the present study when *PROL1* was knocked-out, and 28% of the DEG identified in the previous study when *PROL1* was overexpressed cells [11]). As might be predicted, there was reciprocality in the fold-change in DEG when with *PROL1* was overexpressed compared to knocked-out (i.e DEG that have increased levels of expression in LNCaP cells overexpressing *PROL1* cells, have decreased expression in LNCaP cells with *PROL1* knockout, and vice versa, as shown in Supplementary Table 2). For example, brain acid soluble protein 1 (BASP1, which is upregulated in several cancers and has been reported to promote tumor growth [31, 32], is upregulated with *PROL1* overexpression (Log2FC = 6.32; *p*-value, 1.4 × 10^−8^) and downregulated with *PROL1* knockout (Log2FC = –5.07; *p*-value, 3.2 × 10^−60^). Similarly, semaphorin 4G (SEMA4G, which has been suggested as tumor suppressor gene for colorectal cancer [33]) is downregulated with *PROL1* overexpression (Log2FC = –4.8, *p*-value, 9 × 10^−18^) and is upregulated with *PROL1* knockout (Log2FC = 2.6, *p*-value, 1.2 × 10^−13^).

The complete list of 1563 DEG associated with *PROL1* knockdown, as well as the subset of 312 DEG identified as common between *PROL1* knockdown and *PROL* overexpression, were submitted to DAVID for ontological analysis. The DAVID database recognized 1305 of the 1563, and 271 of the 312 DEG, as unique and identifiable genes. Complete results of gene ontology analysis in biological functions and disease are shown in Supplementary Tables 3,4,5. As shown in Supplementary Table 3, there was significant overrepresentation of DEG in ontological groups related to cancer, including PrCa. Overall, *PROL1* knockout resulted in modulated expression of 72 genes associated with cancer (13 of these genes were also represented in the subset of DEG common between *PROL1* knockout and overexpression) (Supplementary Table 6).

In previous studies it has been demonstrated that overexpression of *PROL1* in LNCaP cells regulates genes involved in aniogenesis and tumor blood supply [11]. This led to the hypothesis that *PROL1* contributes to the development of PrCa by promoting the vascularization of developing tumors, overcoming the hypoxic barrier. This observation was confirmed in the present studies on LNCaP cell-lines with *PROL1* knockdown, where there was significant overrepresentation of DEG in ontological groups related to aniogenesis and tumor blood supply (Table 2A and Supplementary Table 4). Even in the small subset of DEG in common between *PROL1* knockout and *PROL1* overexpression (312 DEG), these ontological groups were overrepresented, often with a greater level of statistical significance (Table 2B and Supplementary Table 5). These two separate investigations, generating mutually supportive evidence, provides a high level of confidence that *PROL1* regulates pathways related to angiogenesis and blood supply to the tumor.

Our analysis also identified an unreported association between *PROL1* expression and the regulation of genes involved in cell motility and migration (Table 2A,2B). Given that increased cell motility and migration are associated with PrCa invasion and metastasis, we conducted experiments to determine if *PROL1* overexpression is associated with modulated motility and migration.

### 3.4 Overexpression of PROL1 in LNCaP cells results in greater cell motility and migration

**FIG. 1.**
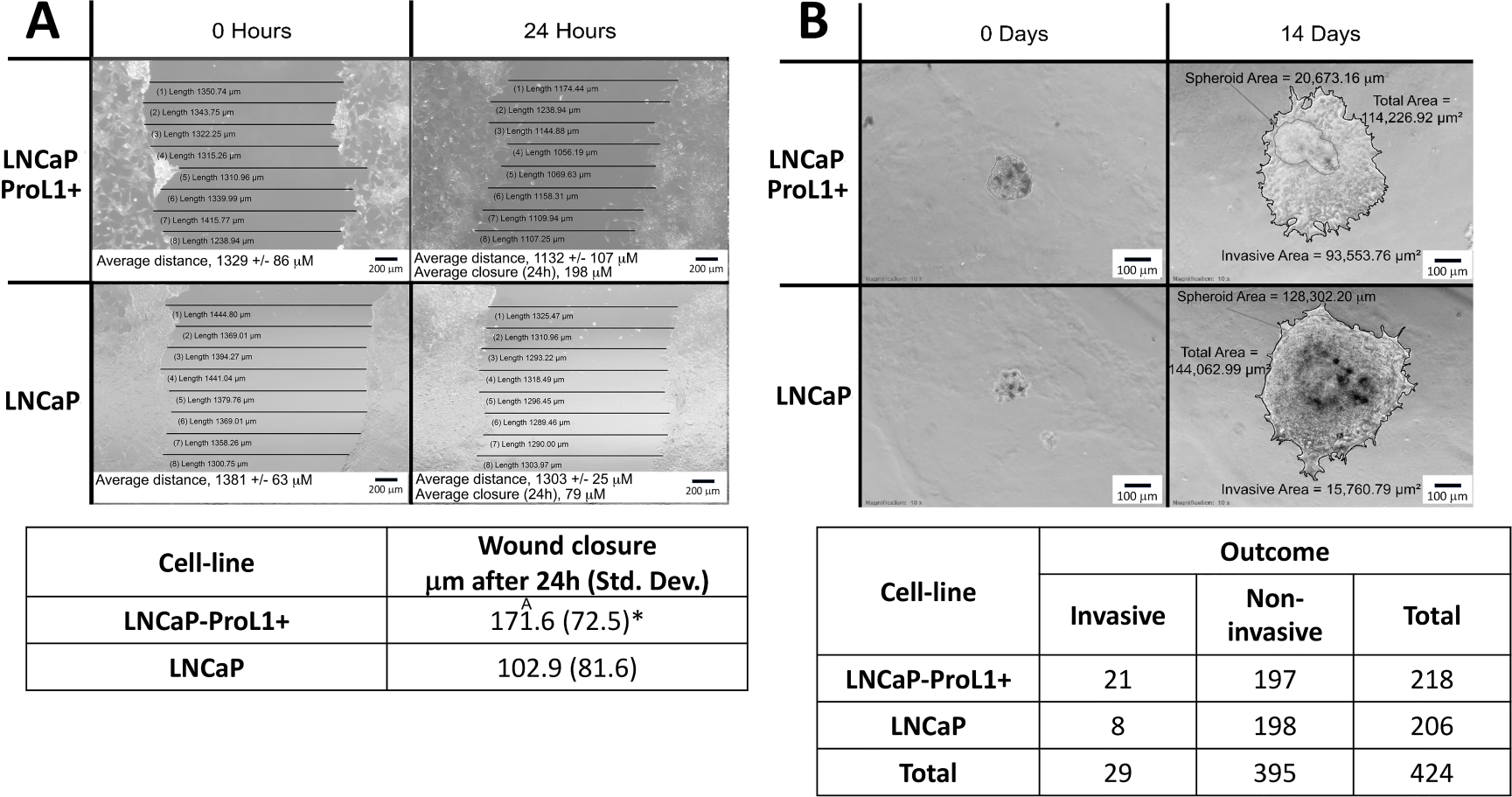
Overexpression of *PROL1* in LNCaP cells results in a more invasive phenotype. (A) A representative wound healing assay for LNCaP-ProL1+ and LNCaP cells is shown (upper panel). The width of the wound was measured at at least eight points per image. Data shown (lower panel) represents the mean rate of wound healing (*μ*m per 24 hours) ± Std. Dev. of five independent experiments (with measurements performed in triplicate for each experiment). * = *p*-value < 0.001. (B) A representative 3D spheroid invasion assay for LNCaP-ProL1+ and LNCaP cells is shown (upper panel). Spheroids were determined to be invasive if a 2D layer of cells was observed growing around the spheroid. Data shown (lower panel) represents the total number of invasive and noninvasive spheroids formed by LNCaP-ProL1+ and LNCaP after 2-weeks. LNCaP-ProL1+ cells generated significantly higher invasive according to the Fisher’s exact test (*p* < 0.0001).

The regulation of genes involved in cell motility and migration by *PROL1* described above, led us to determine if *PROL1* overexpression in LNCaP cells results in changes in phenotype indicative of greater motility and migration. Using a wound healing assay, we demonstrate that that LNCaP-ProL1+ cells have a 1.7-fold increase in the mean rate of wound healing compared to LNCaP (from approximately 103 to 172 mm per 24 hours, Fig. 1A). This increase in cell motility is comparable to results from wound healing assays that have been used to support a role for other genes in promoting invasiveness [34–36].

In addition, we performed a 3D spheroid invasion assay on both LNCaP and LNCaP-ProL1+. As shown in Fig. 1B, there was a significant increase in invasiveness rate in the LNCaP-ProL1+ compared to control LNCaP cells (2.6-fold, *p* = 0.01). This increase is comparable to results from other spheroid growth assays that have been used to support a role for other genes in promoting invasiveness [37–39].

## 4. Discussion

The studies presented here demonstrate that *PROL1* expression is essential for development of xenografted tumors in mice injected with a castration sensitive, human PrCa cell-line (LNCaP). Our global gene expression analysis confirmed a previously reported role for *PROL1* in regulating pathways in angiogenesis and blood supply [11]. In addition, *PROL1* was also identified as a regulator of pathways involved in cell motility and migration. *In vitro* assays confirmed overexpression of *PROL1* in LNCaP cells results in greater cell motility and migration. Overall, our studies add to the increasing body of evidence that modulated *PROL1* expression is associated with PrCa and provides mechanistic insights in to its role in tumor development and progression.

Evidence for a role of opiorphin in cancer has been increasing ever since the publication in 2008 of a rank aggregation analysis to identify common genes which have modulated expression across different cancer types [4]. In the aggregated list of top-50 genes, 36 had been previously been implicated in cancer (often multiple cancers), with *PROL1* a member of the other group of 14 genes, which were suggested as potential novel cancer genes deserving of further scrutiny. Based on the observation that cancer is often associated with modulated neutral endopeptidase (NEP) activity, and that the peptide products of opiorphin genes act as a potent NEP inhibitors, a review article in 2015 also suggested opiorphin may play a role in cancer development [40]. Since the publication of these reviews, several reports have described an association between opiorphin expression and cancer; including breast cancer [3, 5], oropharyngeal squamous cell carcinoma [6, 7], head and neck cancer [8, 9], hepatocellular carcinoma[10] and more recently, PrCa [11]. In the abence of an effective antibody for direct detection of opiorphin protein expression, all of the reported studies, as well as those reported here, are limited to demonstrating an association between opiorphin gene expression and cancer.

The mouse xenograft model is widely used as an animal model of PCa development [41–45], and our studies demonstrate that *PROL1* expression is essential for the development of tumors from injected LNCaP cells. Male mice injected with LNCaP and LNCaP-ProL1+ cells develop tumors, whereas mice injected with LNCaP-ProL1– cells do not. However, our *in vitro* studies demonstrate that under normal culture conditions, neither knockout or overexpression of PROL1 in LNCaP cells impacts growth rate. Therefore, failure of male mice injected with LNCaP-ProL1– cells to develop tumors is not an effect of *PROL1* on growth rate, but rather, the absence of an adaptive response allowing injected cells to establish and grow tumors.

To develop a hypothesis for the role played by *PROL1* in tumor growth, we considered published research on the biological activity of opiorphins and the molecular pathways regulated by *PROL1* described in the present study. Physiological studies have shown that opiorphin directly regulates vascular smooth muscle tone with dysregulated opiorphin expression associated with several pathophysiology’s involving blood flow, such as hypertension[46], erectile dysfunction[47] and priapism[48]. At the molecular level, opiorphin has been shown to be a master regulator of hypoxic response pathways in smooth muscle cells [13, 14], and in the present studies we confirmed that in PrCa cells, *PROL1* expression regulates pathways involved in angiogenesis and blood supply. Based on these observations, we hypothesize that *PROL1* mediates an adaptive response to the hypoxic environment that develops as tumors grow, by activating pathways increasing tumor blood supply.

In addition, the present studies provide evidence that *PROL1* may be also involved in the progression of localized PrCa towards a more invasive, metastatic and castration resistant cancer. Cell motility is a critical step in the progression to metastatic disease, and our global gene expression studies demonstrate that *PROL1* regulates molecular pathways related to cell motility. Although there was no significant affect by *PROL1* overexpression in 2D cell migration assays in Boyden chambers (data not shown), in wound closure and 3D spheroid growth assays, we confirmed that LNCaP-ProL1+ has significantly greater cell motility and migration than its parental cell-line. The difference to the 2D assay is potentially because the positive effects of *PROL1* on cell motility and migration are only observed when cells face a environment more akin to an environment present in a tumor, in vivo, and the 3D spheroid assay is generally considered a better tool to model the phenotypic and cellular heterogeneity, as well as microenvironmental aspects, of tumor growth *in vivo* [49].

Levels of *PROL1* also appear to modulate the androgen-sensitivity of LNCaP cells. Although our in vitro studies demonstrate growth rate under normal culture conditions, when cells are grown in media in which FBS is replaced by charcoal stripped FBS (mimicking an androgen-free environment) LNCaP-ProL1+ cells show significant, 8-fold faster growth than LNCaP-ProL1– cells, suggesting overexpression of *PROL1* results in reduced androgen-sensitivity. Further evidence that *PROL1* is involved in the progression of PrCa cells from an androgen-sensitive to a castration resistant phenotype was provided through xenograft studies in castrated male mice. Remarkably, castrated mice injected with LNCaP-ProL+ cells, in contrast to mice injected with LNCaP, developed tumors.

One of the goals of our research has been to document the role of *PROL1* in regulating ontological groups of genes that may be involved in growth and development cancer (such as those involved in hypoxia, angiogenesis, cell motility and migration, etc). Although global gene expression analysis by techniques such as RNA-seq may have a relatively poor correlation with the change of that specific gene at the protein level (about 40% of genes show both differential level of expression at the mRNA and protein level [50], when changes in ontologic groups of genes with differential expression at the mRNA level are compared with ontologic groups of proteins with differential expression, then the correlation is robust (mean r = 0.71) [51]. Therefore, based on other studies, the correlation with ontological groups with differential protein expression would be predicted to be robust.

A limitation of our approach to identify the most significant ontological groups of genes regulated *PROL1*, is that we cannot rule out the involvement of individual genes involved in additional mechanisms of PrCa growth and development (in addition to hypoxia, angiogenesis, cell motility and migration). In future studies, where perhaps specific therapeutic targets are proposed based on data on changed expression level of a specific gene identified through global gene analysis, confirmation at the protein levels would be important. In addition, our studies have focused on the role of *PROL1* in just one androgen-sensitive cell-line LNCaP. Although LNCaP is one of the most commonly utilized androgen-sensitive cell-lines used in in PrCa preclinical models [41], and are commonly the only cell-line in initial studies, in future studies the rigor of our findings would be improved if similar effects were found in additional PrCa cell-lines.

## 5. Conclusions

Overall, our studies add to the growing body of literature that opiorphin-encoding genes play a role in cancer development and progression. They are essential for establishment of PrCa tumors in mouse xenograft studies, and our evidence supports a possible role in progression towards a more invasive, metastatic and castration resistant PrCa. Targeting opiorphin expression or down-stream pathways regulated by opiorphins are potentially therapeutic strategies to prevent PrCa growth and progression.

## Supplementary Material

Supplemental Table S6

Supplemental Table S5

Supplemental Table S4

Supplemental Table S3

Supplemental Table S2

Supplemental Table S1

Supplemental Figure S1

## Figures and Tables

**TABLE 1.** Growth characteristics of the cell-lines used in these studies.

Cell-line	Growth rate (10% FBS) Doubling time in days ± Std. error	Growth rate (10% charcoal-stripped FBS) Doubling time in days ± Std. error

LNCaP	0.81 ± 0.04	3.01 ± 0.06*
LNCaP-ProL1+	0.71 ± 0.08	1.65 ± 0.09*
LNCap-ProL1−	1.35 ± 0.04	13.2 ± 0.04*

* = a significant decrease in the growth rate of cell-line in charcoal-stripped compared to complete FBS, *p*-value < 0.005.

**TABLE 2A.** Gene ontology analysis of differentially expressed genes resulting from *PROL1* knockout in LNCaP cells: angiogenesis/tumor blood supply/cell migration associated.

Overrepresented ontological group	GO identifier	# Represented genes (1563. submitted, 1305 recognized)	Fold-enrichment	*p*-value

Angiogenesis/ tumor blood supply (DAVID_BP_all)				
Blood circulation	0008015	45	1.54	4.23 × 10^−3^
Regulation of blood circulation	1903522	30	1.73	4.44 × 10^−3^
Vasculature development	0001944	52	1.47	4.83 × 10^−3^
Circulatory system development	0072359	74	1.35	6.07 × 10^−3^
Blood vessel development	0001568	49	1.47	6.41 × 10^−3^
Blood vessel morphogenesis	0048514	42	1.48	1.07 × 10^−2^
Angiogenesis	0001525	36	1.50	1.52 × 10^−2^
Smooth muscle contraction	0006939	11	4.80	2.20 × 10^−2^
Regulation of blood pressure	0008217	17	1.71	3.90 × 10^−2^
Regulation of angiogenesis	0045765	20	1.56	5.48 × 10^−2^
Cell Migration Motility (DAVID_BP_all)				
Cell motility	0048870	112	1.46	3.85 × 10^−5^
Cell migration	0016477	98	1.44	2.35 × 10^−4^
Regulation of cell motility	2000145	65	1.53	6.28 × 10^−4^
Positive regulation of cell migration	0030335	45	1.71	1.36 × 10^−3^
Positive regulation of cell motility	2000147	40	1.69	1.38 × 10^−3^

**TABLE 2B.** Gene ontology analysis of differentially expressed genes in common between *PROL1* knockout or *PROL1* overexpression in LNCaP cells: angiogenesis/tumor blood supply/cell migration associated.

Overrepresented ontological group	GO identifier	# Represented genes (312 submitted, 271 recognized)	Fold-enrichment	*p*-value

Angiogenesis/tumor blood supply (DAVID_BP_all)				
Blood circulation	0008015	17	2.46	1.55 × 10^−3^
Regulation of blood circulation	1903522	10	2.44	2.10 × 10^−2^
Vasculature development	0001944	19	2.38	1.73 × 10^−3^
Circulatory system development	0072359	26	2.02	1.09 × 10^−3^
Blood vessel development	0001568	19	2.42	9.09 × 10^−4^
Blood vessel morphogenesis	0048514	15	2.34	7.28 × 10^−3^
Angiogenesis	0001525	12	2.13	2.61 × 10^−2^
Smooth muscle contraction	0006939	5	4.31	2.74 × 10^−2^
Regulation of blood pressure	0008217	7	3.00	2.94 × 10^−2^
Regulation of angiogenesis	0045765	7	2.31	8.26 × 10^−2^
Cell Migration Motility (DAVID_BP_all)				
Cell migration	0016477	27	1.67	1.04 × 10^−2^
Cell motility	0048870	29	1.59	1.35 × 10^−2^

## Abbreviations

CSFBS: charcoal stripped fetal bovine serum
DAVID: database for annotation, visualization and integrated discovery
DEG: differentially expressed gene
FBS: fetal bovine serum
PrCa: prostate cancer
GO: gene ontology
GOC: gene ontology consortium
NEP: neutral endopeptidase
*PROL1* (aka OPRPN): proline rich, lacrimal 1 (human opiorphin encoding gene)
*hSMR3A/B*: human submaxillary gland androgen regulated protein 3, homolog A (human opiorphin encoding gene)
