# Supplementary material for: PROL1 is essential for xenograft tumor development in mice injected with the human prostate cancer cell-line, LNCaP, and modulates cell migration and invasion
Source: J Mens Health. Author manuscript; Available in PMC 2022 May 10. (PMC9089447; doi:10.31083/jomh.2021.131)
Supplement: Supplemental Table S3 [file NIHMS1779046-supplement-Supplemental_Table_S3.docx]

| Supplemental Table S3: Gene ontology analysis of differentially expressed genes resulting from *PROL1* knockout in LNCaP cells: cancer related. | | | |
| --- | --- | --- | --- |
| Overrepresented ontological group: Cancer related (DAVID_GAD_disease) | ***# Represented genes (1563. submitted, 1305 recognized)*** | **Fold-enrichment.** | **P-value** |
| Urothelial cancer | 4 | 12.39 | 2.41x10^-3^ |
| Esophageal Cancer | 9 | 2.45 | 2.88x10^-2^ |
| Breast cancer/prostate cancer | 9 | 2.45 | 4.46x10^-2^ |
| Lung cancer | 44 | 1.32 | 4.91x10^-2^ |
| Colon cancer/rectal cancer | 3 | 7.75 | 5.23x10^-2^ |
| Liver cancer | 6 | 2.82 | 5.82x10^-2^ |
| Testicular cancer | 4 | 4.13 | 6.79x10^-2^ |
| Prostate cancer | 42 | 1.28 | 8.31x10^-2^ |
| Esophageal/head and neck/laryngeal/mouth/pharyngeal cancer | 8 | 2.1 | 8.38x10^-2^ |
| Recurrent and secondary squamous cell head and neck cancer | 4 | 3.6 | 9.24x10^-2^ |
| Colon cancer | 4 | 3.64 | 9.24x10^-2^ |
| Squamous cell esophageal cancer | 7 | 2.21 | 9.34x10^-2^ |
