# Supplementary figures and images for: PROL1 is essential for xenograft tumor development in mice injected with the human prostate cancer cell-line, LNCaP, and modulates cell migration and invasion
Source: J Mens Health. Author manuscript; Available in PMC 2022 May 10. (PMC9089447; doi:10.31083/jomh.2021.131)

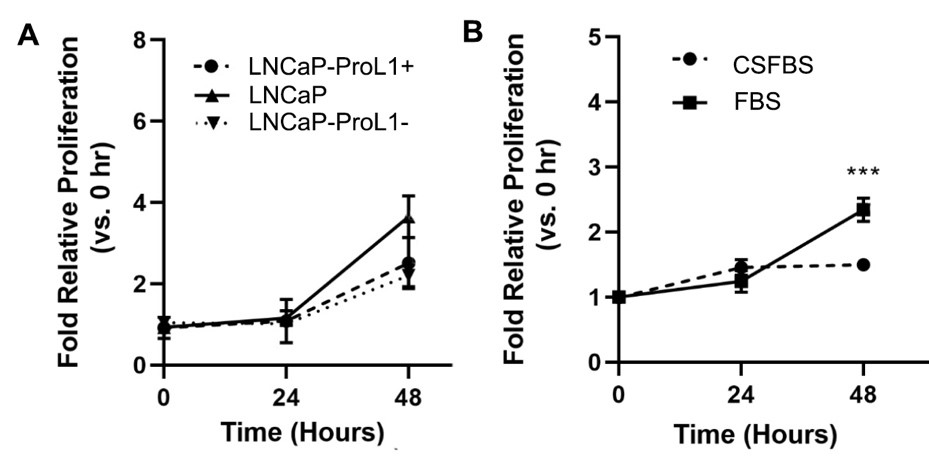

Supplement: Supplemental Figure S1 [file NIHMS1779046-supplement-Supplemental_Figure_S1.jpg]
